# A technical support tool for joint range of motion determination in functional diagnostics - an inter-rater study

**DOI:** 10.1186/s12995-015-0058-5

**Published:** 2015-04-29

**Authors:** Christoph Schiefer, Thomas Kraus, Rolf P Ellegast, Elke Ochsmann

**Affiliations:** Institute of Occupational and Social Medicine, Medical Faculty, RWTH Aachen University, Pauwelsstrasse 30, Aachen, 52074 Germany; Institute for Occupational Safety and Health of the German Social Accident Insurance, Alte Heerstrasse 11153757, Sankt Augustin, Germany; West Saxon University of Applied Sciences, Zwickau, Germany

**Keywords:** Joint range of motion, Inertial measurement units, Functional diagnostics, Human motion capture, Ambulatory field measurement

## Abstract

**Background:**

The examination of joint range of motion (RoM) is part of musculo-skeletal functional diagnostics, used, for example, in occupational examinations. Various examination methodologies exist that have been optimized for occupational medical practice, which means they were reduced to the most necessary and feasible measures and examinations for efficiency and usability reasons. Because of time constraints in medical examinations in occupational settings, visual inspection is commonly used to quantify joint RoM. To support medical examiners, an inertial sensor-based measurement system (CUELA) was adapted for joint RoM examination in these settings. The objective of the present study was to evaluate the measurement tool in functional diagnostics under conditions close to clinical practice.

**Methods:**

The joint RoM of twenty healthy subjects were examined by three physicians, who were simultaneously using the measurement tool. Physicians were blinded to the measurement results and the other physicians. Active RoM was examined on the cervical, thoracic and lumbar spine while passive RoM was examined on the shoulder, elbow, wrist, hip, and knee, resulting in a total of 40 joint examination angles. The means, standard deviations, intraclass correlation coefficients (*ICC*_3,*k*_), and Bland-Altman-Plots were calculated using MatLab for statistical analysis.

**Results:**

Most measurement results were in accordance with expected joint RoMs. All examinations showed an acceptable repeatability. In active RoM examinations, the ICC of inter-rater reliability varied between 0.79 and 0.95. In passive RoM examination the ICC varied between 0.71 and 0.96, except examination angles at the elbow and knee extension (ICC: 0.0-0.77).

**Conclusion:**

The reliability and objectivity of active RoM examinations were improved by the measurement tool compared with examiners. In passive RoM examinations of upper and lower extremities, the increase of objectivity by the measurements was limited for some examination angles by external factors such as the individual examiner impact on motion execution or the given joint examination conditions. Especially the elbow joint examination requires further development to achieve acceptable reliability. A modification in the examination method to reduce the examiner impact on measurement and the implementation of a more complex calibration procedure could improve the objectivity and reliability of the measurement tool in passive joint RoM examination to be applicable on nearly the whole body.

## Background

Musculo-skeletal disorders (MSD) have been the main reason for illness induced work disability in Germany in recent years [1]. To assess MSD, occupational physicians use functional examinations. The examination of joint range of motion (RoM) is part of musculo-skeletal functional diagnostics. According to the neutral-zero-method, RoM is examined by moving the distal segment of a joint from a neutral starting position around a defined rotation axis to the end position [2,3]. The maximum rotation angle is often a measure of the RoM (exceptions are for example: finger-floor distance or chin-jugulum distance). Various examination methodologies exist that have been optimized for occupational medical practice [3,4] and do not aim primarily to provide a precise diagnosis [3], but are qualified to identify functional deficits of employees. For reasons of efficiency and usability, these methodologies were often reduced to the most necessary and feasible measures [3]. Because of time constraints in medical examinations in occupational settings, it is common practice to rely on visual inspection by a physician to quantify the joint RoM in these methods. Additional tools for measurement or to support the subject to adopt neutral start postures are not commonly used. To the authors’ knowledge, there is no study reporting the accuracy of observational joint RoM estimation without measurement support. Holm et al. [5] reported high agreement between goniometer measurements and visual estimates of hip RoM, but also significant differences between both methods. Lowe et al. [6,7] investigated the accuracy of observational posture analysis of ergonomists and reported a misclassification rate of 61%-65%, observing the peak joint angles of shoulder, elbow or wrist during predefined working tasks on a six-category scale of full range of motion.

Even though measurement accuracy is not a major concern of the examination methodologies, objectivity of the assessment could be supported and improved by technical tools. To meet the requirements of efficiency and usability, such a tool needs to be simple to handle and fast in application.

Inertial measurement units (IMU) provide an adequate technology for ambulatory acquisition of human movement, especially joint angles [8,9]. An IMU is a sensor combination of angular rate, acceleration and magnetic field sensors that allows for relative orientation computation in three-dimensional space [10]. IMU technology was already applied to single joint RoM measurements in laboratory environments, for examples at the knee [11], cervical spine [9,12], or shoulder [12-14]. In the vicinity of ferro-magnetic materials, the magnetic field becomes distorted, resulting in an unpredictable orientation error [15]. To avoid external disturbances of the magnetic field sensors, wooden chairs or couches were prepared for examination [12,13,16] or an artificial magnetic field was generated to ensure the homogeneity of the magnetic field in a laboratory environment [12]. However, these environmental requirements are not applicable to occupational medical practice. Other approaches, more independent of environmental conditions, use only angular rate and acceleration information of the IMU [11,17]. The accuracy of continuous orientation estimation of IMUs under ideal conditions is high [18] and in field conditions, too, acceptable accuracy is achievable [17]. Nevertheless, the application of IMUs in functional diagnostics is demanding, as there is no rigid connection between sensors and the bone structure of the body segments; therefore, soft tissue artifacts are to be expected. Furthermore, anatomical calibration is required that allows for conversion of the IMU’s orientation into anatomical angles, according to the recommendations of the International Society of Biomechanics [19,20]. To support the RoM examination in functional diagnostics, we adapted an IMU based measuring system (CUELA [17,21]) to a functional diagnostics tool. CUELA is used for several years now for the ambulatory assessment of physical workloads in occupational settings by recording and analyzing posture and motion data at real workplaces [21]. The developed diagnostics tool should be usable for physicians without special knowledge of IMU technology and oriented towards the fokus ^Ⓒ^ physical examination methodology [3]. This method selection was due to the fact that all examiners were schooled in this method and does not represent any preference for this method. The fokus ^Ⓒ^ method is part of the G46 examination - an occupational medical check-up of the musculo-skeletal system of employees in Germany [22]. This method combines a screening examination with a diagnostic examination. The screening examination is used to recognize functional limitations by applying active RoM examinations. Abnormal screening results lead to the application of the more detailed diagnostic examination. The diagnostic examination provides a systematic method of searching for functional disorders, applying passive RoMexamination.

The objective of the present study was to evaluate the validity, reliability and objectivity of the measurement tool in functional diagnostics under conditions close to clinical practice. The validity is evaluated by comparing the measurement results to expected RoM results from literature. The reliability is evaluated by analyzing repeatability under constant conditions and reproducibility under varying conditions. Finally the objectivity is evaluated by analyzing the rater agreements of measurements and examiner ratings. To the authors’ knowledge, this is the first evaluation study to apply IMUs for joint RoM measurement on nearly the whole body under these conditions.

## Methods

### Study Design

Twenty subjects (age: 37.4 ±9.9 years, height: 176.2 ±8.5 cm, weight 78.8 ±14.8 kg, 14 men and 6 women) volunteered to participate in the study after giving their informed written consent. The local ethical committee of the RWTH Aachen University approved the study. Only healthy subjects without or with minor known functional deficits were recruited. Functional deficits could appear for example in combination with joint diseases, after a bone fracture or after developmental disorders. Subjects with minor functional deficits were recruited depending on a sufficient state of healing and relevance to the examined movements. In the medical history taking, which took place before the examination, the participants declared themselves to be free of musculo-skeletal complaints for at least one week before the examination. Participants unfulfilling these requirements were excluded. Three physicians (with 2-4 years of experience in joint RoM examination) conducted the physical examinations of the subjects. Each physician was supported by one assistant. Examination rooms, equipped with a couch, an IMU-examination-set, and a camera for a video recording of the examination, were assigned to eachexaminer.

### Measurement of joint RoM with IMUs

**Figure 1:**
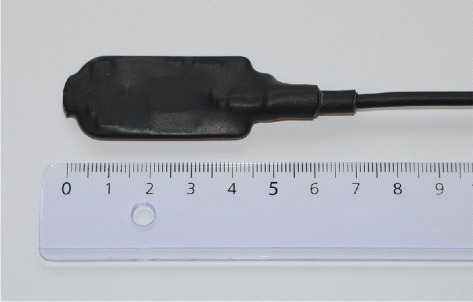
Inertial measurement unit (IMU) of the CUELA system.

To measure the joint RoM, IMUs were placed at the body segments of interest. The IMUs used (Figure 1) are part of the new generation of the CUELA system (CUELA, IFA, Sankt Augustin, Germany [17,21]). Schiefer et al. [17] analyzed the accuracy in three-dimensional orientation estimation of the measurement system. Three IMU-examination-sets, each consisting of 13 IMUs and a notebook for data processing and recording, were used. A self-developed C#.Net software was adapted to act as examination software that interprets the sensor-data in real-time.

Based on the attached sensors and identified sensor positions, the examination software provided a list of applicable RoM examinations. When the assistant chose one joint RoM examination area, the software selected the IMUs corresponding to the joint, the relevant axis of rotation and the expected initial segment posture. Equally to the conventional examination, the subject had to start in a joint specific neutral starting-position and to move the adjacent, distal segment to the end of range of motion [23], while the orientation of both adjacent segments was measured and computed continuously. Depending on the kind of examination movement, the joint angle was calculated by the orientation difference between two IMUs at the adjacent joint segments (e.g. arm and forearm at elbow flexion) or by the orientation difference between the starting and current orientation of one IMU at the distal joint segment (e.g. forearm at shoulder internal/external rotation) in the corresponding plane of motion. The minimal and maximal angle value was assigned to the anatomical nomenclature and stored as the measurement result. The joint angles were calculated using the procedure described by Grood and Suntay [24]. During the examination, the software generated an examination sheet containing all screening results, which were automatically imported into an MS-Access database.

A simplified IMU to segment calibration was used, based on the defined neutral start posture, to initialize the IMU orientation at the beginning of each examination. The procedure is comparable to standard anatomical positions [25], but used only the gravity vector for inclination initialization and the expected segment orientation instead of the magnetometer information for heading. The real-time visualization of the subject’s movement by the examination software allowed to detect whether the calibration was sufficient or not. During examination, the examiner could proceed as usual and did not need to handle a measurement device, as the IMUs are fixed to the segments. Examiners and assistants had a brief introduction to the handling of the measurement tool and the software.

### Examination procedure

**Figure 2:**
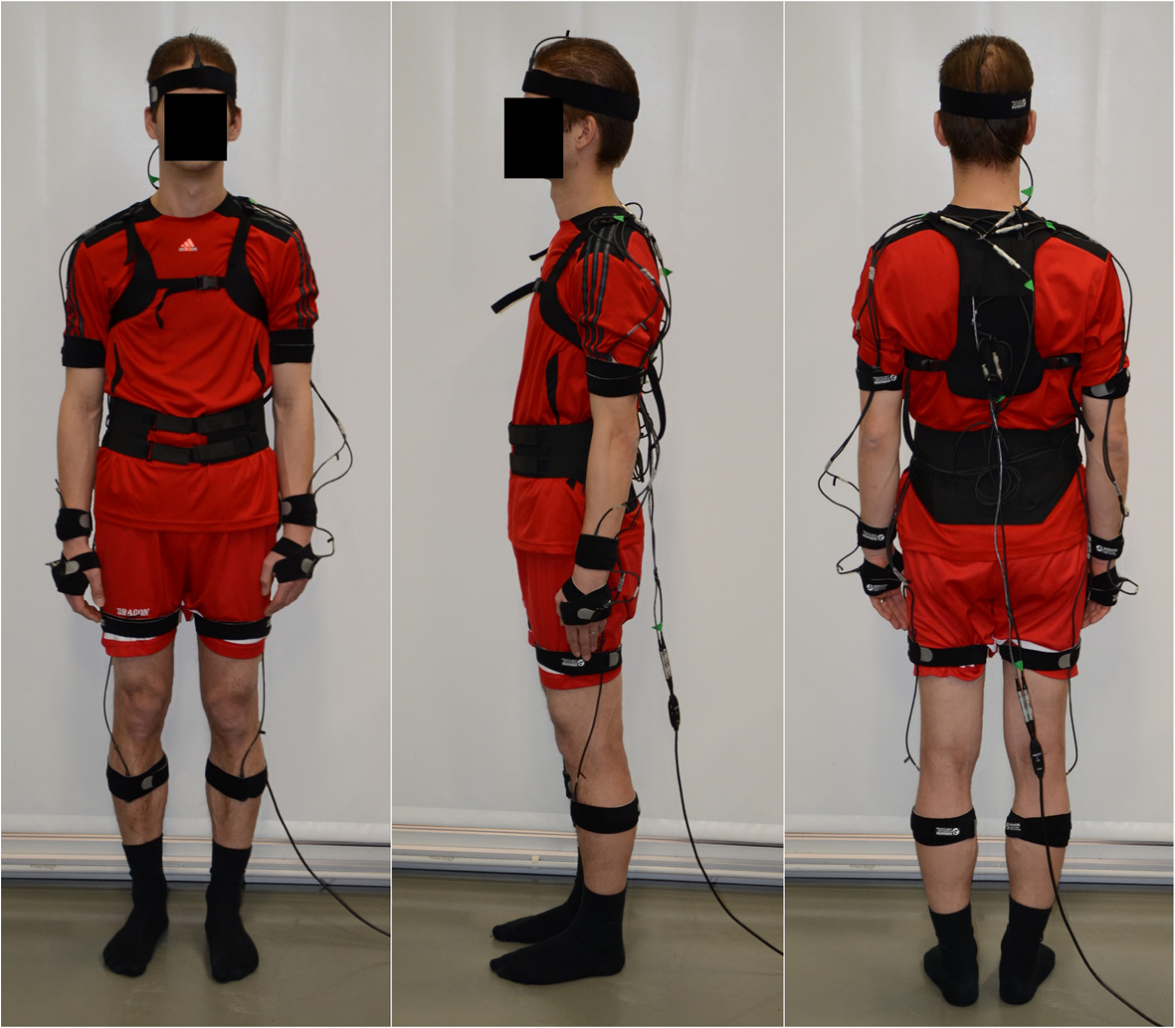
Setup of the IMU based CUELA system. The IMUs were placed on forehead, back, upper arms and forearms, hands, and on upper and lower legs. A hip belt and a harness, carrying the body sensor network infrastructure, fixed the IMUs on the back while elastic velcro straps fixed the IMUs to the extremities.

The IMUs were placed on the forehead, on the back at the level of L5/S1 and Th4 [9], laterally on the upper arms and on the forearms close to the wrist, on the dorsum of the hand, laterally on the upper legs and frontally on the lower legs. In the case of legs and upper arms, the sensors were placed in the middle of the segments. A hip belt and a harness, carrying the body sensor network infrastructure, fixed the IMUs on the back while elastic velcro straps fixed the IMUs to the extremities (Figure 2). In each examination room, the assistant helped to equip the participant with the sensor system. Equipping the participants with sensors took 5 to 8 minutes while removal took 1.5 to 2.5 minutes. After the preparation of the participants, the examination could start immediately. To avoid warming or training effects [26], each examination motion was practiced three times [9,12,16,27,28]. After warming up, each joint angle examination/measurement was repeated five times. The examiner assessed each repetition while it was measured by IMUs simultaneously. The assistant operated the screening software and wrote the screening results of the physicians on an examination sheet. One group of joints was examined actively (cervical, thoracic and lumbar spine) while the other group was examined passively (shoulder, elbow, wrist, hip, and knee).

All participants were examined by the three physicians and their assigned IMU-examination-set. To keep the physical state of the participants as constant as possible, all three examinations of each participant took place within one day, one directly after the other in random order. The examiners were blinded to the measurement results of the IMUs and to the otherexaminers.

### Data analysis

All measurements and examination data were collected in an MS-Access database and prepared for statistical analysis with Matlab (Mathworks, Inc.). Comparator basis was the mean of the five examination repetitions by the physicians and the simultaneous IMU measurements.

For evaluation of the validity of measured and observed RoM, the mean value of the five measurements/examinations was compared to a collection of suggestions for normal RoM values in healthy adults ([2], pp. 472) and the expected minimum RoM angles that healthy adults should have when the fokus ^Ⓒ^ methodology is being applied [23].

The intra-rater repeatability under constant conditions was evaluated based on the mean standard deviation within the five measurement repetitions.

Intraclass correlation coefficients (ICC) were calculated to evaluate the inter-rater reliability of the measurements compared to the examiner ratings. The *ICC*_3,*k*_ type was selected for quantification of reliability, as the same raters measured all participants and the mean of repeated measures was compared [29]. An *ICC*_3,*k*_ value ≥0.8 was interpreted as an acceptable level of reliability, according to Jordan et al. [12] or Berryman Reese et al. [2], pp.40.

For further analyses of inter-rater agreement between examiners and measurement tool, Bland-Altman-Plots [30,31] were used. The mean of two raters is assigned to the X-axis and the difference between raters is assigned to the Y-axis. The mean difference (MDiff) is represented by a blue line and indicates a systematic over- or underestimation of this rater combination, if different from zero. The upper and lower 95% limits of agreement (LOA) are represented by red lines.

## Results

### Validity of measurement

Table 1 shows the mean joint RoM results of all subjects, separated into examiner ratings and corresponding measurement by the measurement tool. Most examination results matched the expected RoM of the reference values of healthy adults. In case of cervical spine flexion, the measurement results (61 ^∘^-65 ^∘^) exceeded the RoM expectations of 50 ^∘^ while the examiners differed in their ratings (36 ^∘^-72 ^∘^). In external and internal shoulder rotation, the measurement results (92 ^∘^-111 ^∘^) and examiner ratings (88 ^∘^-105 ^∘^) exceeded the RoM expectations of 90 ^∘^. The measured mean elbow extension of 16 ^∘^ to 22 ^∘^ corresponding to examiner 1 exceeded expectations, while the mean examiner ratings were plausible. The measured mean elbow supination (47 ^∘^-76 ^∘^) and pronation (68 ^∘^-86 ^∘^) angles were below the expectations (90 ^∘^), while the examiner observed the expected values.

**Table 1:** **Mean and standard deviation in brackets of joint RoM [** ^**∘**^ **], separated by examiner**

**Joint**	**Examination angle**	**Meas1**	**Ex1**	**Meas2**	**Ex2**	**Meas3**	**Ex3**	**Reference1 [** 2 **]**	**Reference2 [** 23 **]**
*Active RoM - screening examination*									
Cervical spine	Rotation, L	75 (8)	74 (9)	66 (9)	63 (7)	69 (11)	76 (6)	80	70
	Rotation, R	74 (9)	74 (9)	69 (9)	60 (9)	70 (10)	76 (8)	80	70
	Extension	57 (12)	39 (14)	53 (13)	43 (12)	53 (12)	44 (15)	60	45
	Flexion	65 (9)	36 (5)	61 (12)	46 (11)	62 (8)	72 (12)	50	45
	Lateral flexion, L	42 (10)	33 (9)	37 (8)	37 (6)	39 (10)	45 (11)	45	45
	Lateral flexion, R	39 (9)	32 (8)	36 (8)	37 (9)	36 (10)	44 (10)	45	45
Thoracic and lumbar spine	Sideways rotation, L	43 (5)	40 (7)	41 (6)	31 (3)	45 (9)	33 (10)	30	30
	Sideways rotation, R	43 (8)	38 (7)	43 (7)	30 (4)	42 (6)	34 (9)	30	30
	Lateral bending, L	33 (9)	34 (7)	33 (7)	28 (6)	33 (6)	43 (12)	30	30
	Lateral bending, R	31 (7)	37 (7)	31 (6)	29 (5)	32 (8)	44 (11)	30	30
*Passive RoM - functional diagnostic examination*									
Shoulder	External rotation, L	100 (12)	105 (14)	95 (9)	91 (4)	100 (15)	97 (15)	90	90
	External rotation, R	111 (13)	104 (12)	95 (11)	91 (6)	99 (15)	99 (11)	90	90
	Internal rotation, L	109 (13)	91 (6)	94 (11)	90 (4)	96 (15)	93 (12)	70	90
	Internal rotation, R	97 (11)	88 (9)	92 (12)	88 (8)	94 (16)	88 (11)	70	90
Elbow	Extension, L	22 (6)	10 (2)	5 (3)	0 (1)	12 (6)	2 (3)	0	10
	Extension, R	16 (5)	11 (3)	4 (3)	0 (1)	8 (6)	1 (2)	0	10
	Flexion, L	138 (11)	131 (10)	144 (10)	139 (13)	140 (11)	158 (13)	140	150
	Flexion, R	137 (9)	132 (13)	140 (12)	136 (15)	136 (13)	156 (12)	140	150
	Pronation, L	70 (20)	94 (4)	86 (9)	90 (1)	77 (11)	90 (2)	80	90
	Pronation, R	68 (11)	94 (4)	77 (10)	89 (3)	78 (7)	90 (1)	80	90
	Supination, L	47 (18)	87 (6)	68 (16)	89 (4)	76 (14)	90 (5)	80	90
	Supination, R	61 (18)	86 (6)	67 (14)	89 (3)	71 (14)	90 (6)	80	90
Wrist	Extension, L	78 (17)	94 (8)	76 (12)	89 (3)	83 (16)	91 (5)	70	60
	Extension, R	80 (15)	92 (11)	79 (14)	89 (3)	84 (18)	89 (5)	70	60
	Flexion, L	86 (16)	81 (7)	82 (12)	87 (8)	78 (20)	83 (13)	80	60
	Flexion, R	85 (13)	80 (7)	80 (12)	85 (7)	77 (13)	83 (15)	80	60
	Abduction, L	27 (6)	19 (6)	21 (5)	18 (6)	21 (7)	22 (10)	20	30
	Abduction, R	24 (6)	15 (6)	19 (7)	17 (8)	21 (7)	19 (6)	20	30
	Adduction, L	35 (8)	34 (8)	32 (6)	34 (10)	35 (12)	32 (7)	30	40
	Adduction, R	34 (6)	38 (7)	37 (6)	32 (7)	32 (10)	32 (7)	30	40
Hip ^∗^	Flexion, L	128 (10)	143 (11)	120 (11)	125 (7)	105 (15)	142 (20)	120	130
	Flexion, R	128 (11)	142 (10)	124 (10)	125 (9)	117 (13)	143 (22)	120	130
	Lateral rotation, L	57 (8)	54 (7)	52 (11)	37 (10)	48 (8)	44 (10)	35-40	50
	Lateral rotation, R	60 (13)	53 (5)	52 (11)	43 (8)	51 (10)	43 (10)	35-40	50
	Medial rotation, L	49 (13)	41 (10)	33 (11)	26 (10)	33 (9)	26 (11)	35-40	40
	Medial rotation, R	50 (15)	39 (10)	33 (11)	26 (10)	37 (13)	25 (12)	35-40	40
Knee ^∗^	Extension, L	5 (3)	6 (3)	1 (1)	0 (0)	1 (2)	0 (0)	0	5
	Extension, R	5 (3)	7 (2)	1 (2)	0 (0)	1 (1)	0 (2)	0	5
	Flexion, L	145 (10)	144 (10)	142 (8)	139 (9)	133 (8)	162 (11)	140-145	150
	Flexion, R	141 (9)	138 (8)	144 (8)	140 (7)	135 (9)	161 (10)	140-145	150

Given is the observation of the examiner and the corresponding measurement result by the IMUs of all subjects (n =20). The last two columns provide reference values of healthy adults from literature. (^∗^In case of hip and knee assessment only n=12 subjects could be taken into account).

### Intra-rater repeatability

Table 2 shows the mean of the standard deviation within the five immediate examination/measurement repetitions. In case of active movement the mean SD of measurements is in a range of 1.35 ^∘^ to 2.86 ^∘^ while, in case of passive movement, the range is higher from 0.98 ^∘^ to 4.87 ^∘^. The examiners achieved a comparable low mean standard deviation below 1.23 ^∘^.

**Table 2:** **Mean of standard deviation [** ^**∘**^ **] within five immediate examination repetitions of joint RoM examination, separated by measurements and examiners**

**Joint**	**Examination**	**Mean SD**	**Mean SD**
	**angle**	**measurements**	**examiners**
Active RoM - screening examination			
Cervical spine	Rotation	2.60	0.86
	Extension	2.54	1.09
	Flexion	2.86	0.42
	Lateral flexion	1.98	1.23
Thoracic and	Sideways rotation	2.48	0.64
lumbar spine			
	Lateral bending	1.35	0.61
Passive RoM - functional diagnostic			
examination			
Shoulder	External rotation	4.36	1.16
	Internal rotation	4.87	1.04
Elbow	Extension	3.01	0.30
	Flexion	4.06	0.55
	Pronation	4.76	0.20
	Supination	3.94	0.29
Wrist	Extension	3.58	0.54
	Flexion	4.23	0.48
	Abduction	2.97	0.36
	Adduction	2.93	0.63
Hip	Flexion	2.35	0.95
	Lateral rotation	3.13	0.44
	Medial rotation	3.10	0.46
Knee	Extension	0.98	0.05
	Flexion	2.03	0.64

### Inter-rater reliability

Table 3 contains the ICC values of the measurement results in comparison to the examiner ratings of each examined joint RoM angle. In active RoM examination, most measurements (ICC: 0.79-0.95) showed acceptable reliability. The cervical spine rotation to the left side (ICC: 0.79) was marginally below the acceptable limit. Compared to the examiner ratings, all active RoM examinations using the measurement tool achieved a higher reliability level.

**Table 3:** **Intraclass correlation coefficients** ***I*** ***C*** ***C***_**3,*k***_ **comparing the reliability of examiner ratings and measurements**

**Joint**	**Examination**	**ICC**	**ICC**
	**angle**	**(Measurements)**	**(Examiners)**
Active RoM - screening examination			
Cervical spine	Rotation, L	0.79	0.42
	Rotation, R	**0.83**	0.59
	Extension	**0.89**	0.51
	Flexion	0.83	0.37
	Lateral flexion, L	0.93	**0.86**
	Lateral flexion, R	**0.95**	**0.85**
Thoracic and	Sideways rotation, L	**0.80**	0.69
lumbar spine			
	Sideways rotation, R	**0.90**	0.74
	Lateral bending, L	**0.90**	**0.83**
	Lateral bending, R	**0.92**	0.79
Passive RoM - functional diagnostic			
examination			
Shoulder	External rotation, L	0.71	0.76
	External rotation, R	**0.86**	**0.81**
	Internal rotation, L	**0.88**	0.68
	Internal rotation, R	**0.87**	0.78
Elbow	Extension, L	0.20	0.20
	Extension, R	0.59	0.16
	Flexion, L	0.69	0.50
	Flexion, R	0.77	0.77
	Pronation, L	0.48	0.53
	Pronation, R	0.43	0.56
	Supination, L	0.39	**0.83**
	Supination, R	0.70	0.71
Wrist	Extension, L	**0.88**	0.65
	Extension, R	**0.90**	**0.82**
	Flexion, L	**0.90**	**0.89**
	Flexion, R	**0.86**	**0.84**
	Abduction, L	0.79	0.61
	Abduction, R	**0.89**	**0.83**
	Adduction, L	**0.82**	0.74
	Adduction, R	**0.87**	0.79
Hip ^∗^	Flexion, L	**0.87**	0.63
	Flexion, R	**0.92**	**0.80**
	Lateral rotation, L	**0.87**	**0.90**
	Lateral rotation, R	**0.96**	0.77
	Medial rotation, L	**0.91**	**0.95**
	Medial rotation, R	**0.93**	**0.90**
Knee ^∗^	Extension, L	0.0	0.0
	Extension, R	0.61	0.0
	Flexion, L	**0.82**	**0.81**
	Flexion, R	**0.84**	0.71

Acceptable reliability values were bold printed. (n=20 subjects; ^∗^In case of hip and knee assessment only n =12 subjects could be considered).

In passive RoM examination, most measurements at shoulder, wrist, hip and knee achieved acceptable reliability (ICC: 0.71-0.96). All measured examination angles at the elbow showed slight to moderate reliability (ICC: 0.20-0.77). For extension of elbow and knee, especially on the left side, poor reliability (ICC: 0.0-0.2) was observed. Comparing the measurements and examiner ratings, in 73% of passive examination angles the measurement tool showed a higher ICC compared to the examiner ratings.

### Inter-rater agreement

**Figure 3:**
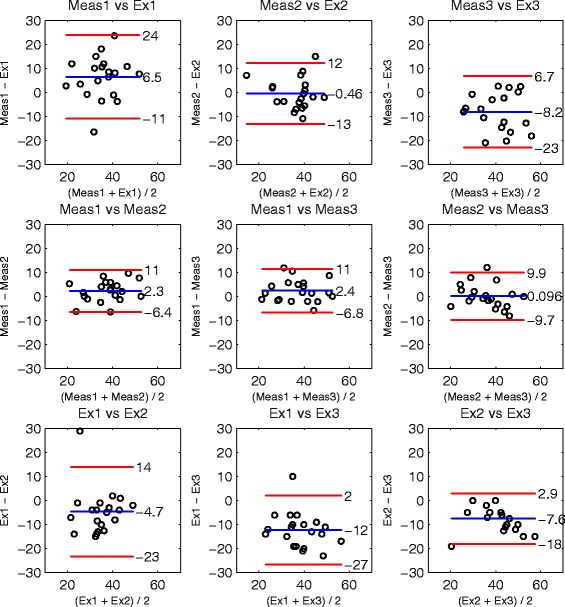
Cervical spine, active lateral flexion to the right side. Bland-Altman-Plots of joint RoM angles [ ^∘^] analyzing agreement of examiners and corresponding measurements. In the first row the examiner rates are compared to the measurement result, in the second row the three measurements and in the last row the examiner rates. The mean difference is indicated by a blue line. The upper and lower limits of agreement (95%) are indicated by red lines.

**Figure 4:**
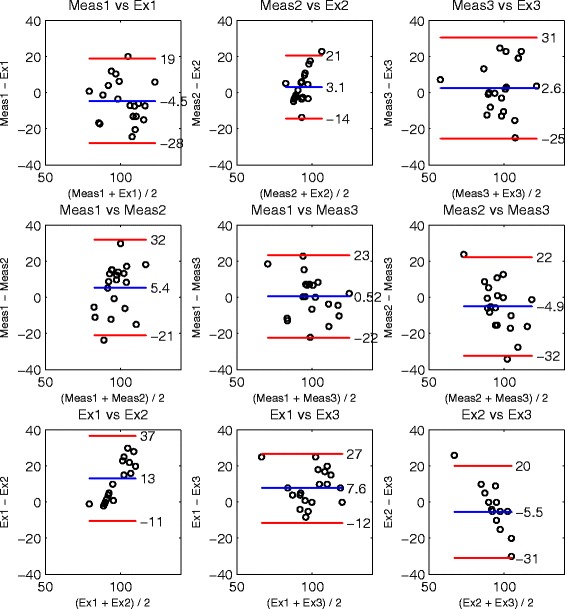
Left shoulder joint, passive external rotation. Bland-Altman-Plots of joint RoM angles [ ^∘^] analyzing agreement of examiners and corresponding measurements.

**Figure 5:**
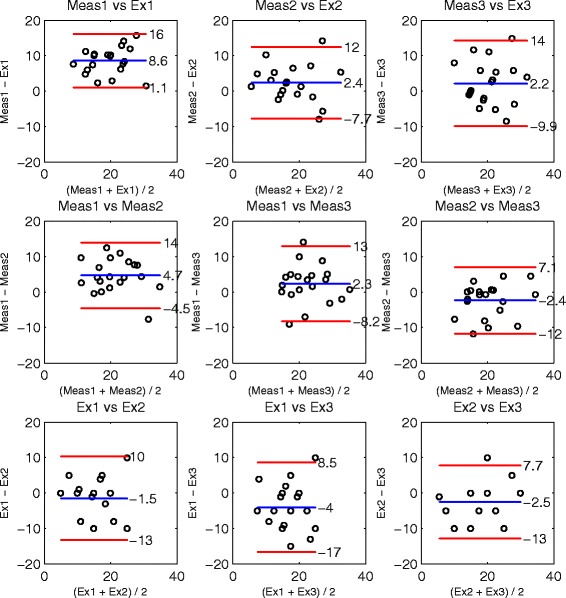
Right wrist joint, passive abduction. Bland-Altman-Plots of joint RoM angles [ ^∘^] analyzing agreement of examiners and corresponding measurements.

Bland-Altman-Plots [30] allow a more detailed analysis of reliability and rater agreement. As a total of 40 joint angles were assessed, only three examples (two good cases and one bad case) are shown in Figures 3, 4 and 5. The Bland-Altman-Plots were arranged in a set of 3x3 plots, showing in the first row the agreement of the examiners versus their measurement results, in the second row each combination of measurement results and in the last row each combination of the examiner ratings.

#### Active RoM examination

Figure 3 shows the agreement for the examination of active lateral flexion of the cervical spine to the right side. This case represented the best case of active RoM examination concerning the achieved ICC level. The first row indicated a trend of the first examiner for underestimation while the third examiner tended to overestimate the RoM in comparison to the measurement. The second examiner showed good agreement with the measurements with an MDiff close to zero. Comparing the three measurement results in the second row indicated agreement with a low systematic difference below 2.4 ^∘^. The examiners showed a higher systematic difference of up to 12 ^∘^. The LOA of the measurement results (≤11^∘^) were smaller compared to the examiner results (≤27^∘^). When inspecting the other Bland-Altman-Plots of active RoM examination, in most cases a reduction of MDiff and LOAs was observed in the measurements. Defining an absolute MDiff below 5 ^∘^ as an acceptable level of systematic difference, an acceptable MDiff level was achieved by 90% of measurements and 33% of examiner comparisons.

#### Passive RoM examination

The agreement of passive external rotation of the left shoulder joint is depicted in Figure 4 and represents a bad case that did not achieve an acceptable ICC level. In this example, the trend of over- or under-estimation of the examiner combination reflected in the corresponding measurement results, indicating the examiner’s influence on the measurement. This effect was observable for shoulder external rotation, lateral and medial rotation of the hip and extension of elbow and knee. In other passive joint examinations (Figure 5, acceptable ICC level) the effect of examiner influence on the measurement was not observed in this form. The inspection of the other passive examination plots indicated in 67% of the cases either an acceptable level of MDiff of measurement or at least a reduction of MDiff by the measurements in comparison to the examiner.

## Discussion and conclusion

In this study, a measurement tool which was designed to support physicians’ medical examinations of the musculo-skeletal system in an occupational setting was evaluated and compared to the examiner ratings. We found that the overall validity and the intra-rater repeatability of the measurement tool were acceptable. Furthermore, inter-rater reliability was acceptable for active examinations, while passive examinations were associated with an influence of the respective examiner on the measurement. These results will be discussed in the following passages.

### Validity

A valid anatomical joint angle calculation of the measurement tool was seen in most applied examination angles, when compared to reference values from the literature, which are defined as a range of expectable RoM angles, caused by differences in the examination methods. The used measurement tools of the references were in most cases inclinometer or goniometer [2,23]. Deviations of measurement results from expected values occurred for cervical spine flexion, shoulder rotation, and elbow measurements. The differences in cervical spine flexion could be explained by different reference points for RoM estimation, even though they should have been the same. Wolff et al. [26] used the projected shadow of a pointer mounted on top of the head to measure cervical spine RoM and reported even higher mean flexion angles of 72.5 ^∘^. In external and internal shoulder rotation, examiners and measurement tool agreed in their observation of a higher RoM that would be plausible. The measured pronation and supination RoM angles of the elbow were below expectations, which would possibly be caused by soft tissue artifacts in combination with the examiner holding forearm and hand. As the IMU could not be connected rigidly to the bone structure, the sensor movement on the skin at the wrist was reduced compared to the observable hand movement. Alternatively, the pronation and supination angles were computed and analyzed using the IMU at dorsum of the hand, but showed unexpected high results based on the same effect. Holden et al. [32] observed a displacement of surface mounted sensors of 10 mm translation and 8 ^∘^ rotation at the shank during walking. While moving the subjects segment to the end of RoM, an examiner could touch and displace the IMUs on the skin leading to those soft tissue artifacts, especially at wrist and hand. The deviation in elbow extension of measurement1 could be caused by a slight systematic elbow flexion during the start of measurement by examiner1. As the algorithm computes orientation relative to the starting orientation, a deviation from the neutral posture causes a shift in the resulting measurements.

### Intra-rater repeatability

Analyzing repeatability required constant measurement conditions. This was given for the five examination movement repetitions, as the examiner, the sensor set, the sensor fixation to the human body, the sensor initialization and initial orientation were identical. Under these conditions, the repeatability of the measurements depended only on the ability of the participants, partly in combination with the examiners, to repeat the examination movements in the same way.

The low deviation within the repeated measures indicated a good repeatability of the examination movement by the participants and a precise measurement of the movement by the system. In case of passive examination, a higher but also acceptable standard deviation was observed. This can be explained by having two protagonists being involved in movement execution, leading to a less accurate movement repetition. Another reason is that the examiner could touch the IMUs while handling the subject’s segment, which would lead to an additional random movement of the IMU independent of the subject’s movement.

The examiners tended to keep their rates of the five examination repetitions constant, which would explain the low deviation within the repeated examiner rates. Furthermore most examiners used to rate the RoM angles on a 5 ^∘^ scale instead of using a continuous scale, which may also lead to a reduction of the standard deviation.

### Inter-rater reliability

For inter-rater reliability analyses the previous conditions varied: three different examiners used their assigned sensor set, leading to an individual sensor fixation, initialization and initial orientation.

An improvement in examination reliability under these variable conditions was observable in active RoM examination of cervical, thoracic and lumbar spine. Even though external factors (examiners, sensor sets, fixation to the body, initialization and initial orientation) varied between the examinations, an acceptable level of reliability was achieved by the measurement tool, which was higher compared to the reliability level of the examiner ratings. In the case of active joint examination, the examiners gave only instructions on how to perform the examination movements but did not touch the subjects. For that reason they had little impact on the movement execution and the resulting measurements. Theobald et al. [9] evaluated optimum IMU placement for measuring active cervical spine RoM. Under more constant conditions, they reported intra-rater reliability of repeated measures in a range of ICC:0.70-0.99. Using an electromagnetic tracking system (FASTRAK), Jordan et al. [12] achieved an inter-rater reliability (*ICC*_2,1_) of measuring active cervical spine RoM in a range from 0.61 to 0.89. As the applied procedures and statistical models differ, a direct comparison of the results is difficult.

In the case of passive joint examination the examiners manipulated the subjects’ body segments, while the subjects had to relax their muscles. Now the examiners had a high impact on both the neutral start position and the motion execution and finally on the resulting measurements. Under these conditions, the measurement tool achieved mostly acceptable reliability for the examination of shoulder, wrist, hip and knee. Examining active shoulder RoM, Jordan et al. [12] achieved an inter-rater reliability (*ICC*_2,1_) in a range from 0.68 to 0.75. Nussbaumer et al. [16] analyzed passive hip range of motion in arthrosis patients using an electromagnetic tracking system by one examiner and achieved in a test-retest design a reliability (*ICC*_2,1_) in a range from 0.82 to 0.95. In the examination of the elbow, acceptable reliability could not be achieved. As the analysis of validity showed deviations from expectations in the pronation, supination and extension of the elbow joint, this is also reflected in the results of the reliability analysis. Besides the aforementioned soft tissue effects and the examiner impact, the simplified anatomical calibration procedure could explain these results. Functional calibration procedures exist [11,25] that should improve the reproducibility of anatomical calibration, but there are still challenges in the anatomical calibration of the upper extremities [25]. Furthermore these methods require a higher preparation effort in application and user know how. The poor reliability level of knee and elbow joint extension examination could be explained by the small magnitude of the measured angle compared to the effect of slight variances in the starting position.

### Inter-rater agreement

The analysis of Bland-Altman-Plots indicated an improvement of the agreement, leading to a higher level of objectivity, in active joint RoM examination of the spine by the measurement tool. In passive examination, an increase of the level of objectivity was only particularly observable for the measurement tool. In some cases the trend for over or under-estimation by the examiner was reflected in the measurements. Although the same examination procedure was applied, individual examiner impact on the execution of the examination movements would be the main reason for the lower level of agreement between measurements. Additional auxiliary aid (for example a table on which to place the arm) would help to reproduce the starting conditions for examination [2], which should improve the examination agreement. In passive joint examination not only a measure for the RoM is of interest. The examiner has also the chance for a tactile impression, for example, of the end of range of motion (limited by bone or soft tissue) [3] or warming of an inflamed joint. The measure of active RoM thus is more important than in passive examination.

### Limitations

Limitations in the presented study should be mentioned: First, for organizational reasons eight subjects needed to be examined in their normal clothing. At the lower extremities their normal clothing hindered the subjects in reaching the end of range of motion. The results of these subjects were not taken into account for analyses of the lower extremities, which is marked in the tables. A second limitation is that there was no direct comparison between active and passive examination agreement to assess the examiner’s impact on the same joint examination. As we intended to cover a whole body examination and were limited in time for all examinations, we applied the examinations either one way or the other. In further research both alternatives should be considered. Finally, a limitation not of this study but of the examination method using IMUs is the necessity to start the measurement in the correct starting position of the defined neutral-zero posture. If a subject has a functional deficit and is not able to take the zero-posture (for example a deficit in extending the elbow or knee joint), the measurement tool is not able to detect this deficit, being dependent on the starting conditions.

### Conclusion

To support the joint RoM examination in functional diagnostics we adapted an IMU system to the fokus ^Ⓒ^ physical examination methodology. With comparable low effort in cost and preparation time, the IMU-examination system measures the joint RoM while a physician applies the examination as usual without the need to operate additional measurement tools.

The evaluation of the tool under conditions close to clinical practice was the objective of this study. Acceptable repeatability was observed, and most measurements showed valid results that met expectations. In active RoM examination of cervical, thoracic and lumbar spine, reliability and objectivity between independent measures was improved by the measurement tool compared with the examiner rates. In passive RoM examination of upper and lower extremities, the increase of objectivity by the measurement tool was limited for some examination angles by external factors such as the individual examiner impact on motion execution or the given joint examination conditions. Especially the elbow joint examination requires further development to achieve acceptable reliability and agreement. A modification in the examination procedure to support the reproducibility of the start postures could improve objectivity. The implementation of a more complex anatomical functional calibration procedure of the measurement tool could improve reliability in the measured passive joint RoM to be applicable on nearly the whole body. Both approaches would lead to an increase in examination effort and a reduction in the simplicity of the examination procedure that conflicts the expectations on the examination method of being fast and simple. To keep these costs of improvement low will be an important challenge for further development of the measurement tool to support joint range of motion determination in functional diagnostics.
